# Supplementary material for: A BOOTSTRAP MODEL COMPARISON TEST FOR IDENTIFYING GENES WITH CONTEXT-SPECIFIC PATTERNS OF GENETIC REGULATION
Source: bioRxiv. 2023 Oct 22:2023.03.06.531446. Originally published 2023 Mar 7. Preprint. [Version 2] doi: 10.1101/2023.03.06.531446 (PMC10028853; doi:10.1101/2023.03.06.531446)
Supplement: Supplement 1 [file media-1.pdf]

Additional figures for "A bootstrap model comparison test for identifying genes with context-specific patterns of genetic regulation"

Mykhaylo M. Malakhov[1], Ben Dai[2], Xiaotong T. Shen[3], and Wei Pan[1]

[1]Division of Biostatistics, University of Minnesota
[2]Department of Statistics, The Chinese University of Hong Kong
[3]School of Statistics, University of Minnesota

1 Simulation results for null comparisons

Below are quantile-quantile (QQ) plots of P-values from the DRAB differential regulation test applied to simulated gene expression data and real genotype data from the GTEx v8 data set. Comparisons are between independent simulations of the same tissue, e.g. testing for differential regulation between one simulated brain cortex data set and another simulated brain cortex data set. Training set sample sizes were fixed at 238 and test set sample sizes were fixed at 239, while the choice of test set tissue and the amount of sample overlap between training sets were varied. The same 50 autosomal protein-coding genes were included in each simulation study. The theoretical null distribution (assuming no data set-specific genetic regulation) is plotted as a solid gray line, flanked by 95% confidence bands.

1.1 Trained and tested on whole blood (WHLBLD)

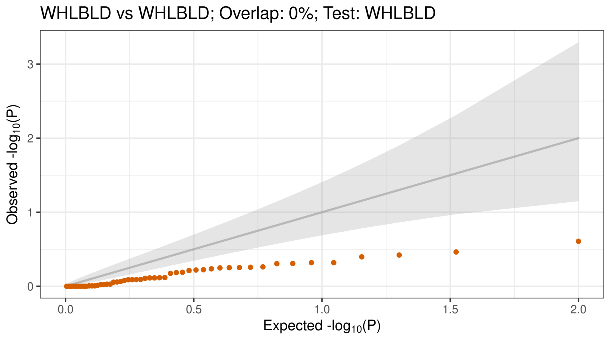

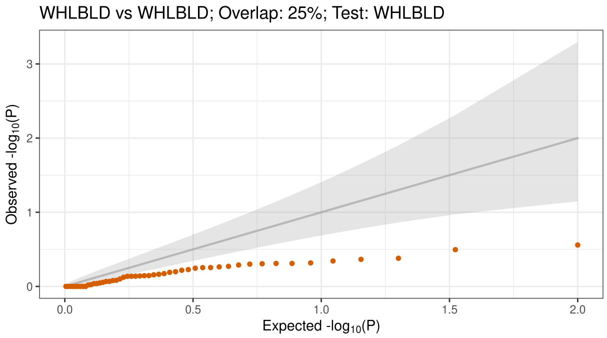

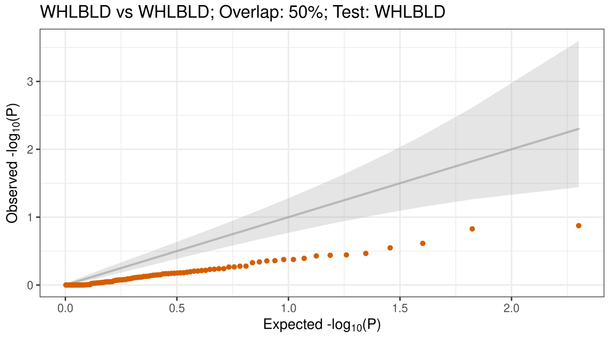

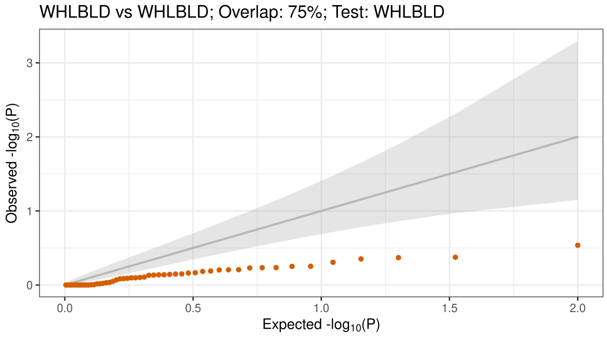

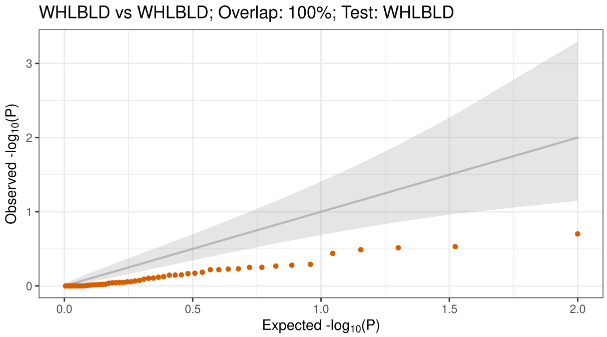

1.2 Trained and tested on brain cortex (BRNCTXA)

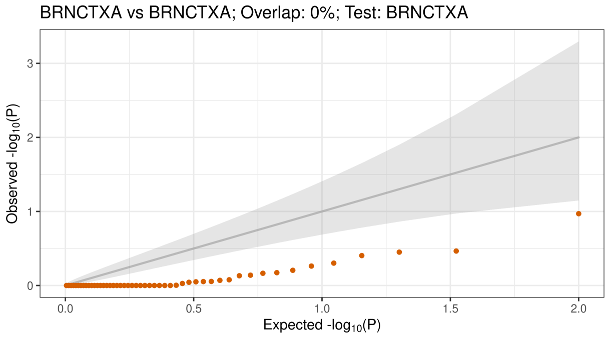

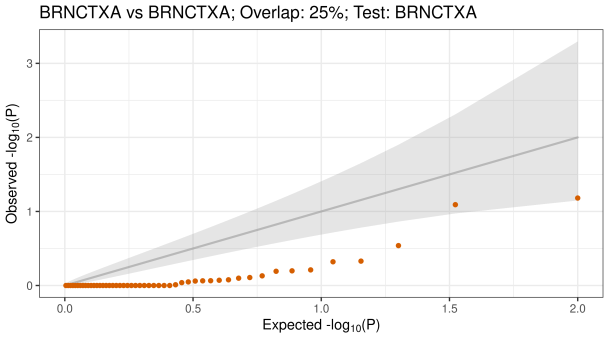

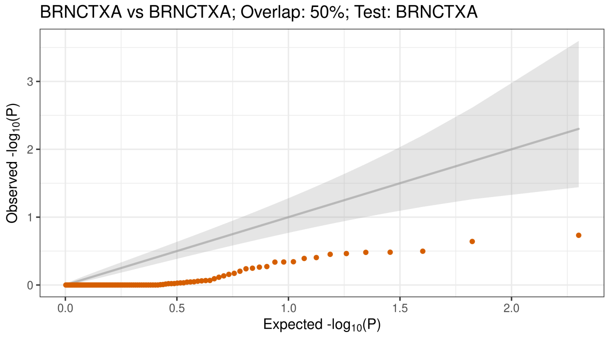

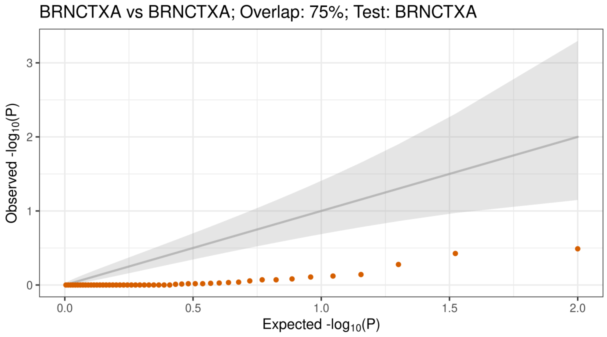

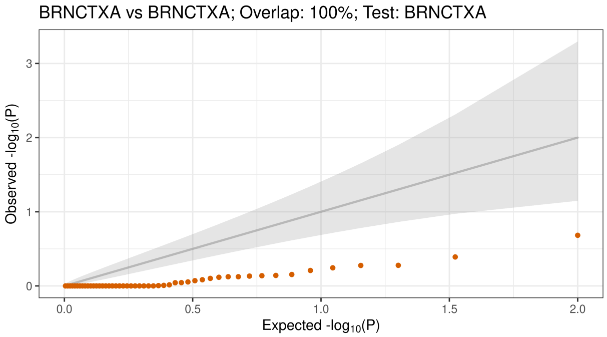

1.3 Trained on brain cortex (BRNCTXA), tested on whole blood (WHLBLD)

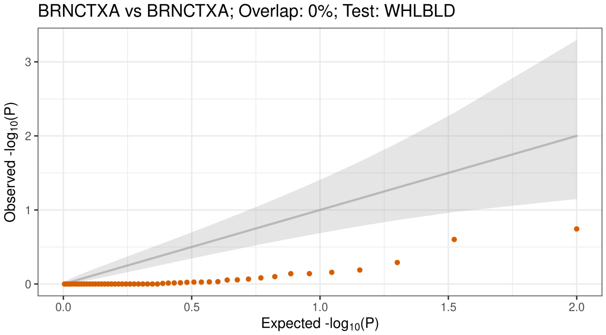

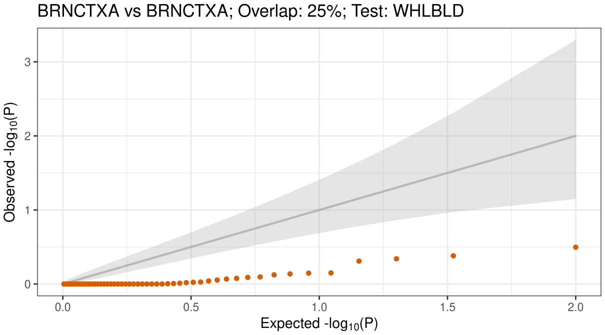

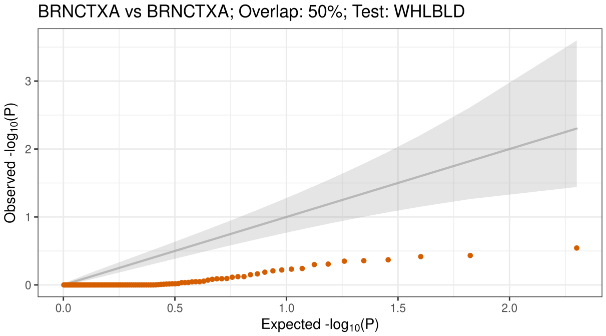

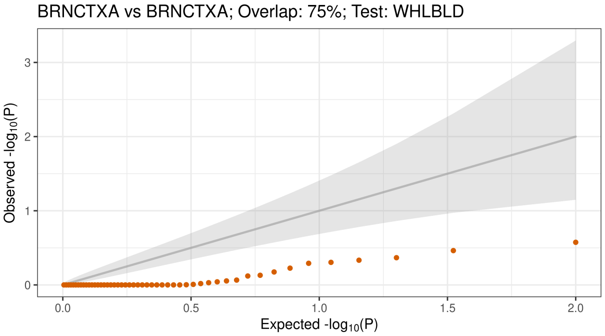

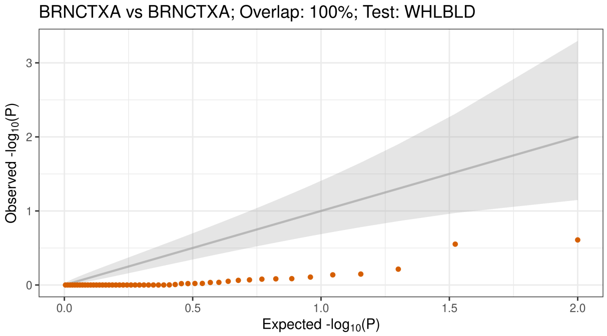

1.4 Trained on whole blood (WHLBLD), tested on brain cortex (BRNCTXA)

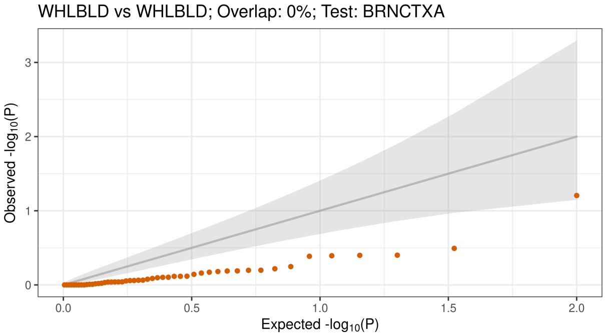

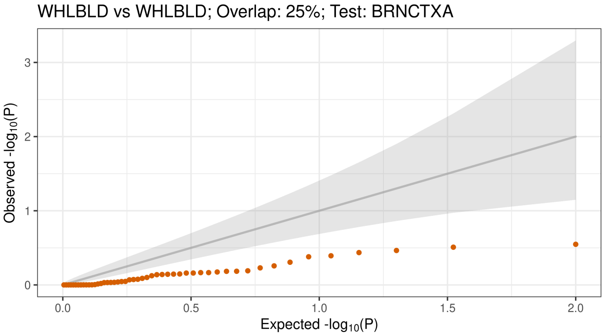

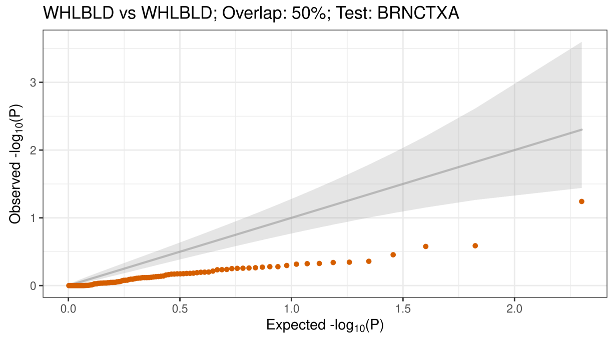

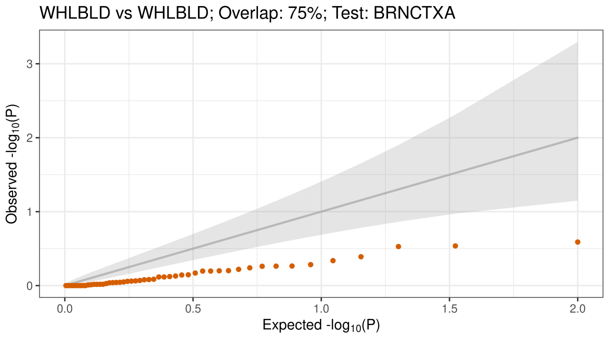

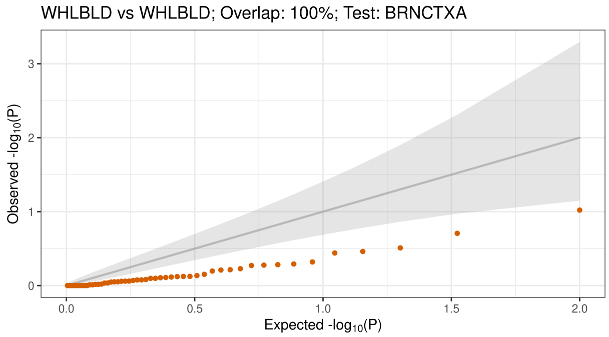

2 Simulation results for power analyses

Below are scatter plots of P-values from the DRAB differential regulation test applied to simulated gene expression data and real genotype data from the GTEx v8 data set. Each comparison is between simulated whole blood expression data and simulated brain cortex expression data. All simulation parameters except those specified in the plot labels were fixed at the following default values: each training set had a sample size of 238, there was no sample overlap between the two training sets, and the test set had a sample size of 239. The same 50 autosomal protein-coding genes from the previous section were included in each simulation study.

2.1 Effect of sample overlap when testing on brain cortex (BRNCTXA)

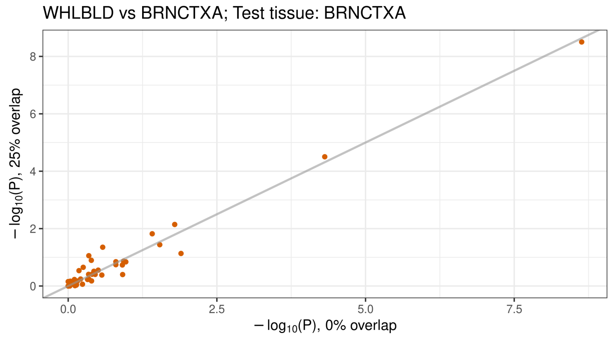

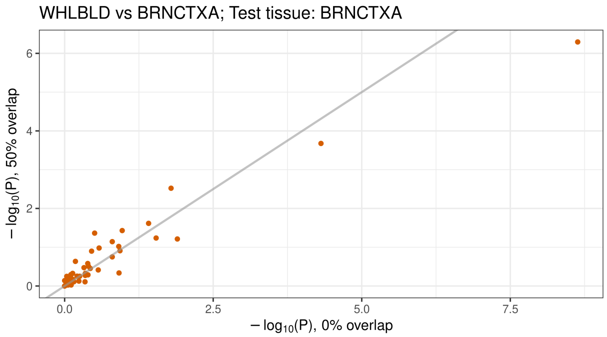

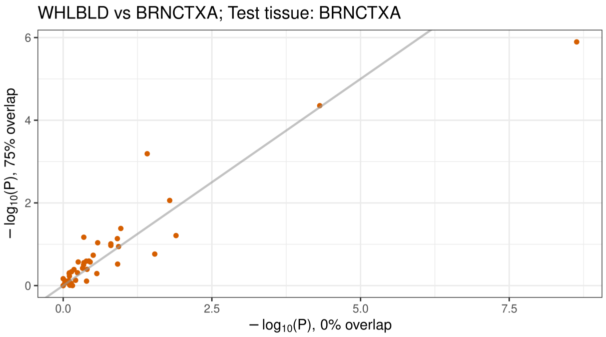

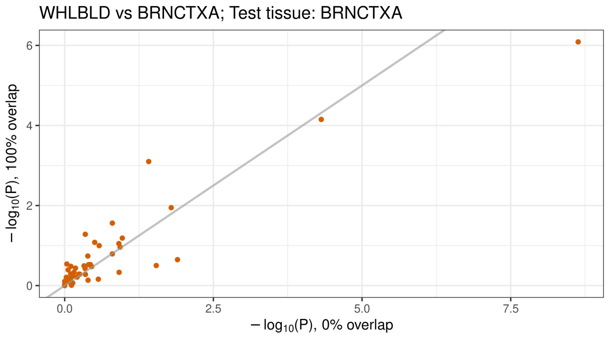

2.2 Effect of sample overlap when testing on whole blood (WHLBLD)

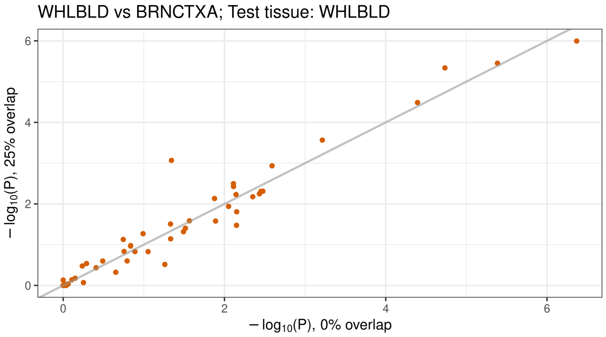

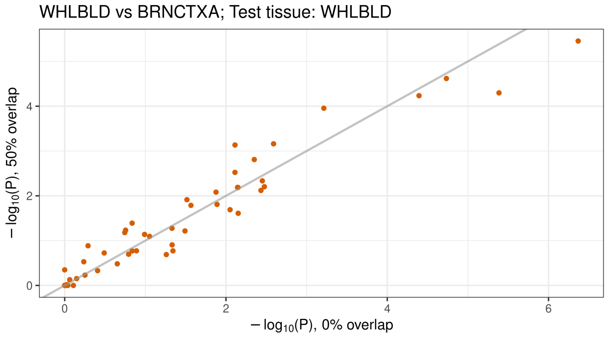

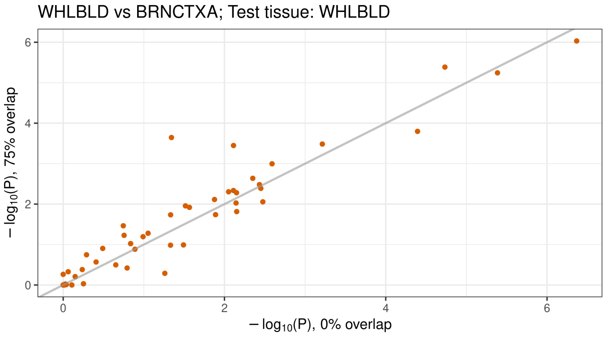

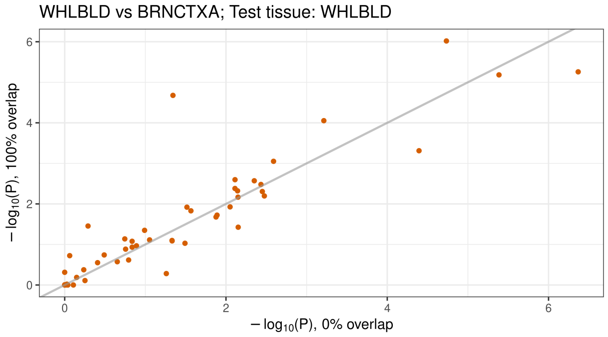

2.3 Effect of test tissue choice

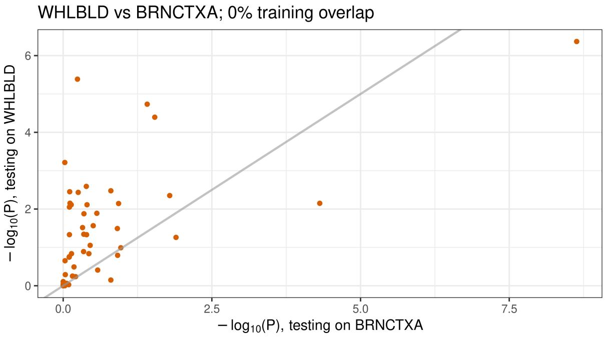

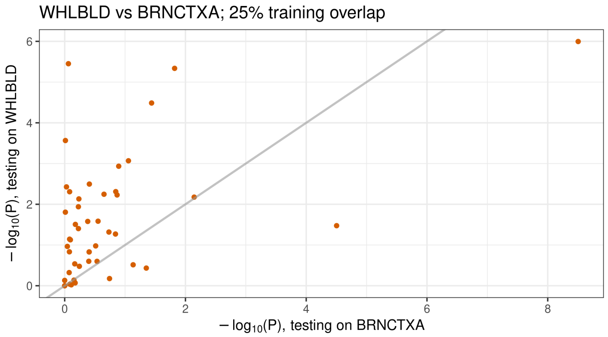

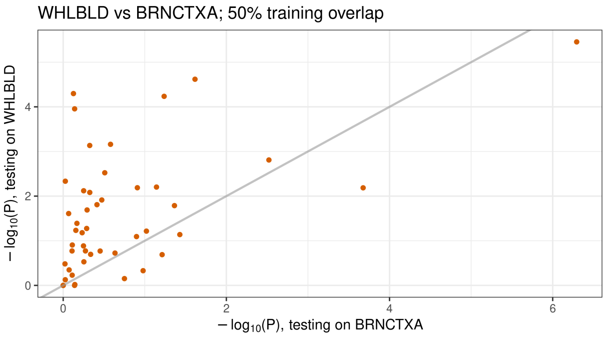

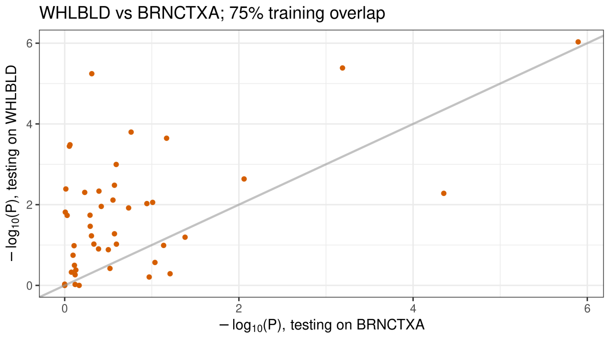

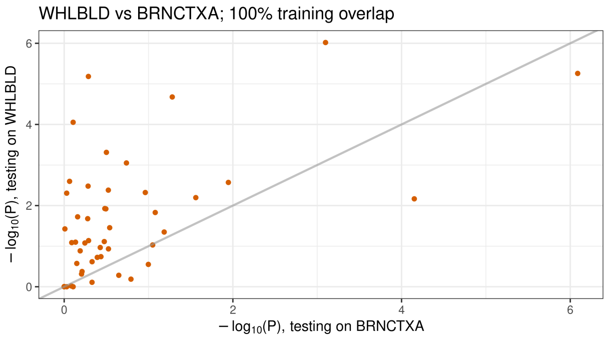

2.4 Effect of test set sample size when testing on whole blood (WHLBLD)

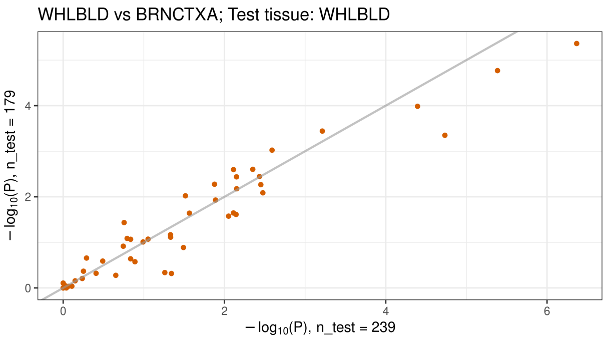

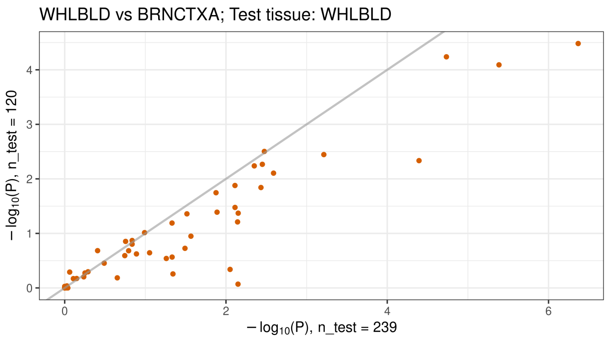

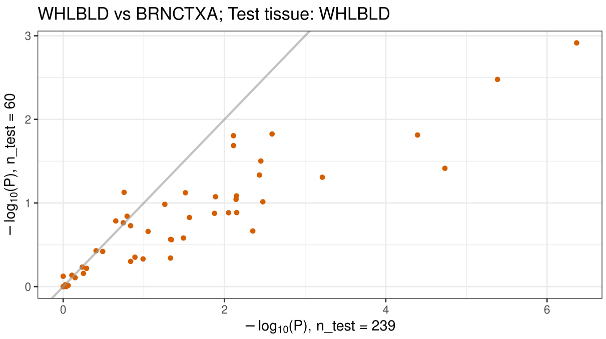

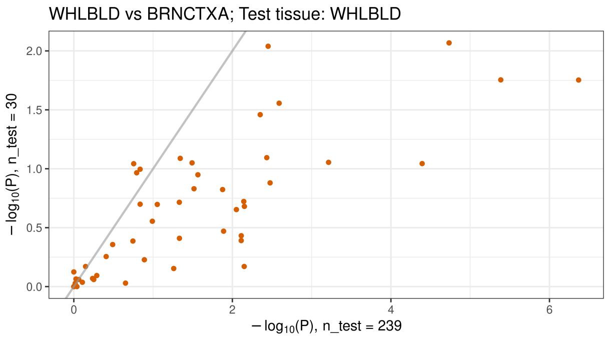

2.5 Effect of test set sample size when testing on brain cortex (BRNCTXA)

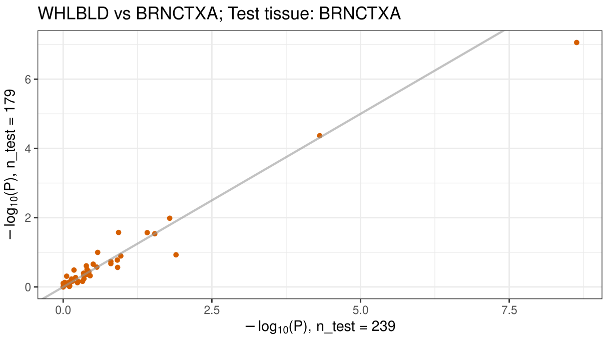

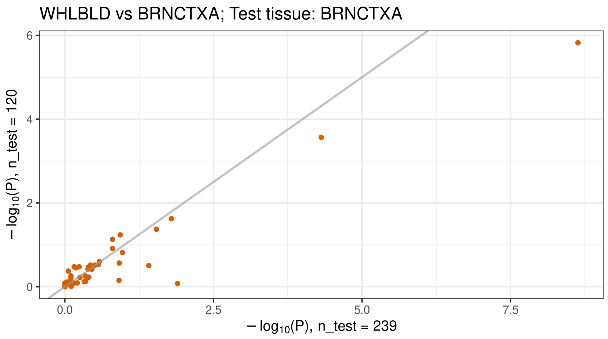

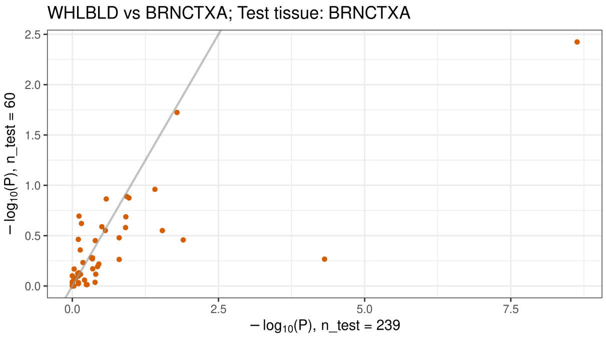

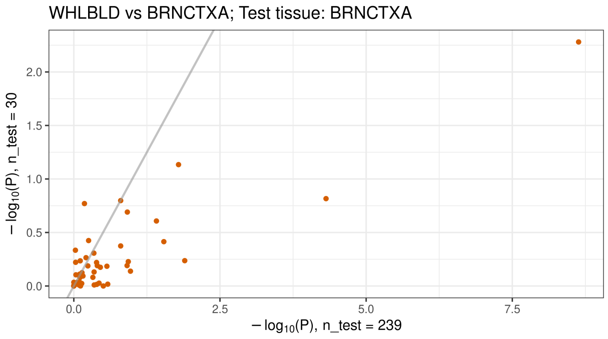

2.6 Effect of training set sample size

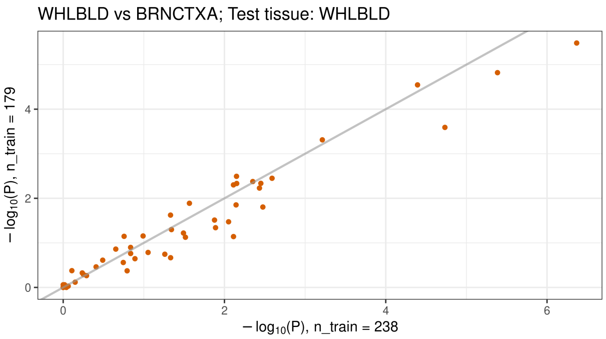

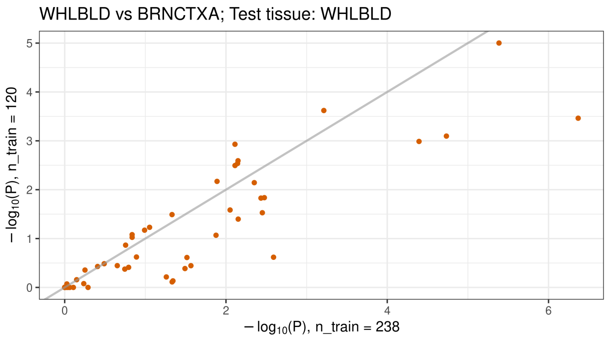

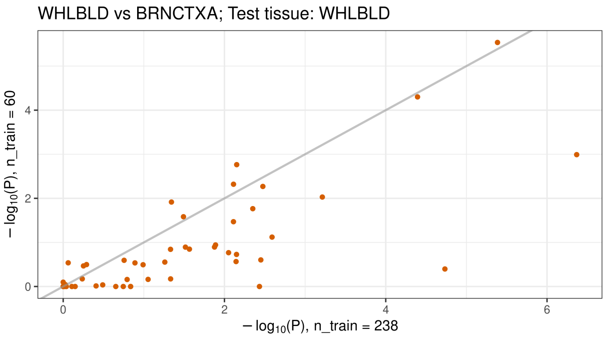

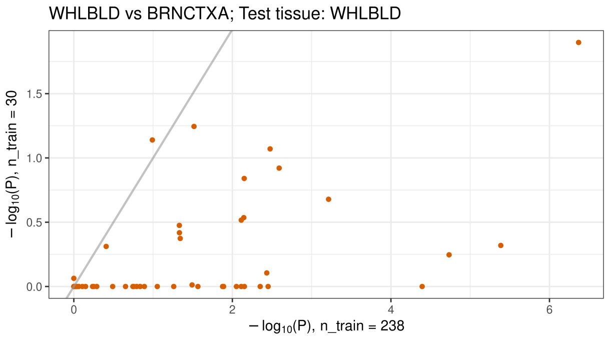

3 QQ plots for all real data tissue pairs

Below are quantile-quantile (QQ) plots of P-values from the DRAB differential regulation test for every tissue pair considered in our real data analysis. All autosomal protein-coding genes were included in the tests. The theoretical null distribution (assuming no tissue-specific genetic regulation) is plotted as a solid gray line, flanked by 95% confidence bands. Definitions for all tissue abbreviations are provided in Table 1 in the main text.

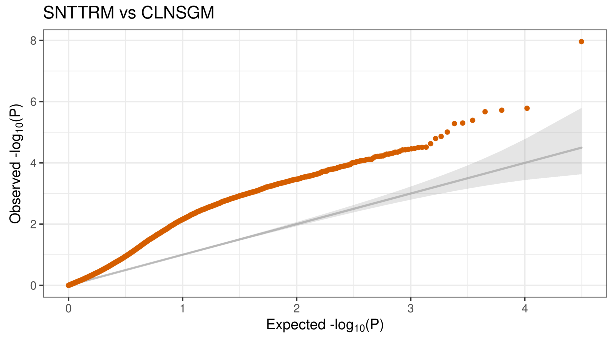

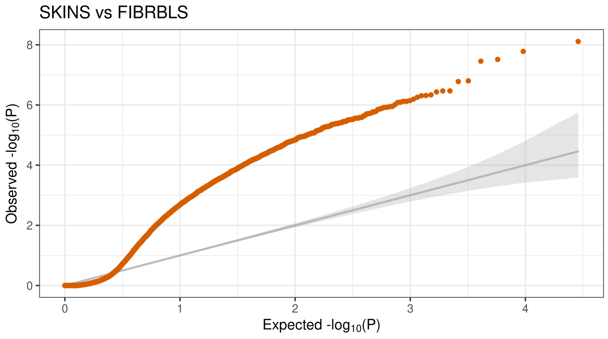

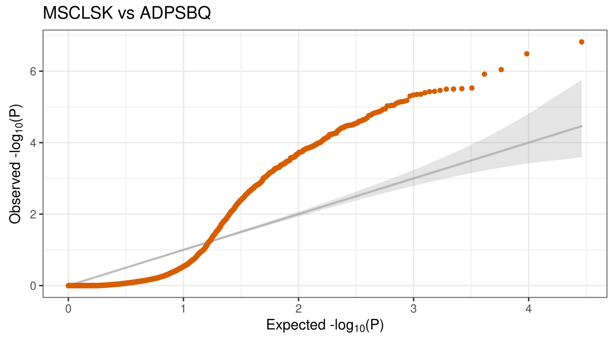

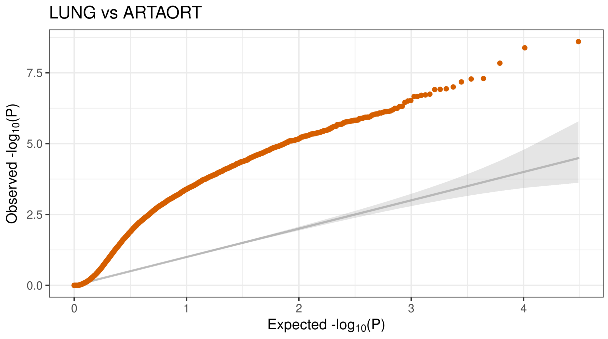

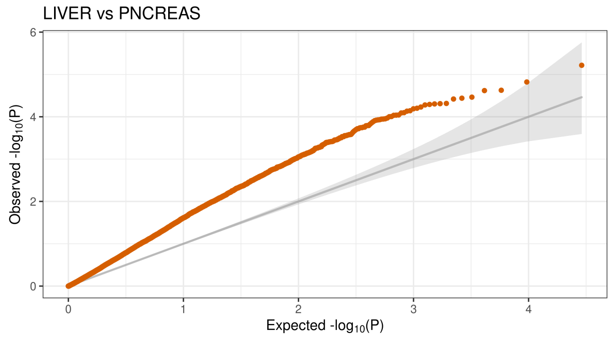

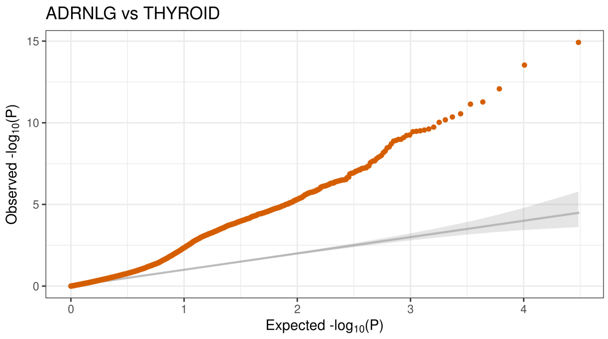

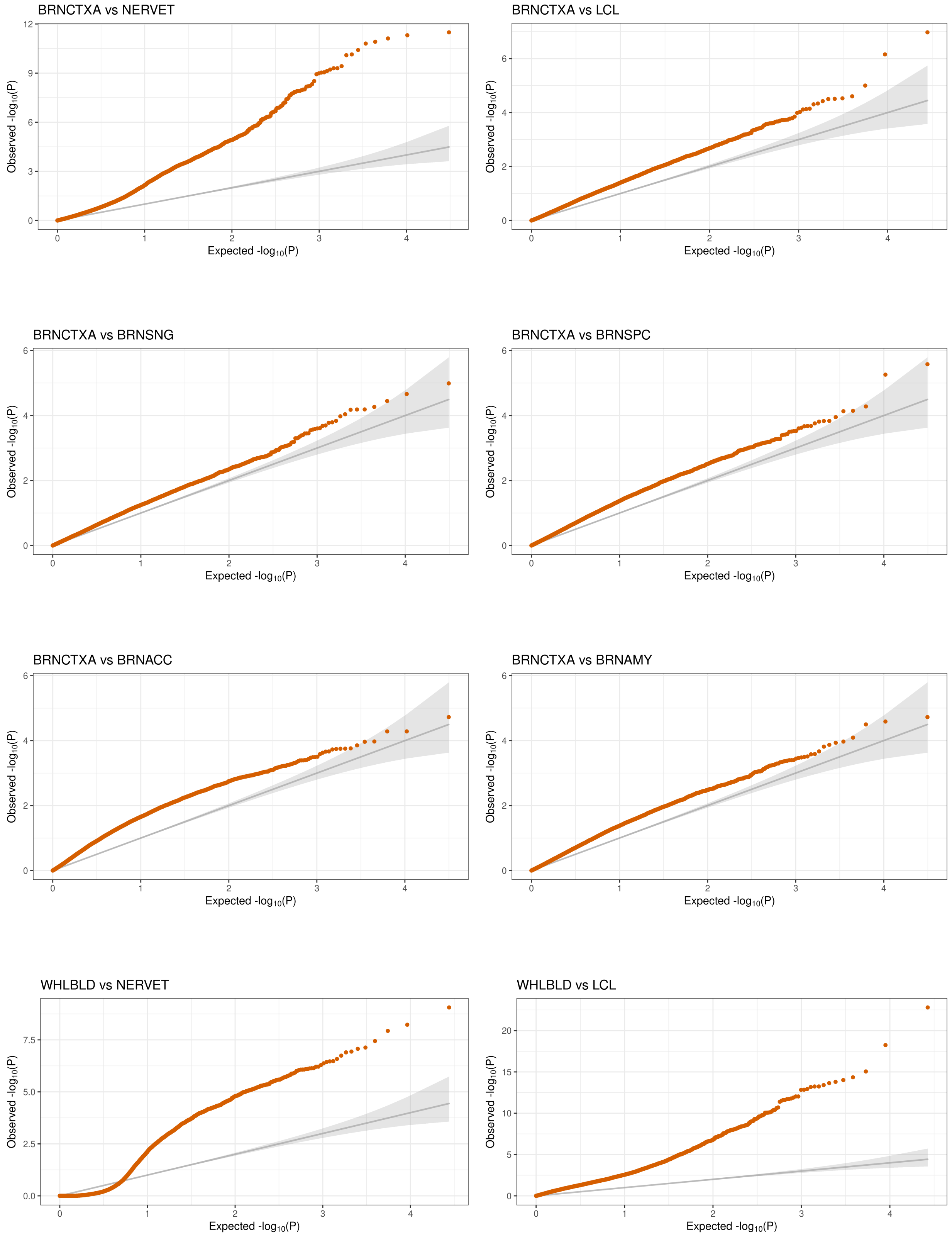
BRNCTXA vs NERVET
BRNCTXA vs LCL
BRNCTXA vs BRNSNG
BRNCTXA vs BRNSPC
BRNCTXA vs BRNACC
BRNCTXA vs BRNAMY
WHLBLD vs NERVET
WHLBLD vs LCL
Observed -log10(P)
Expected -log10(P)

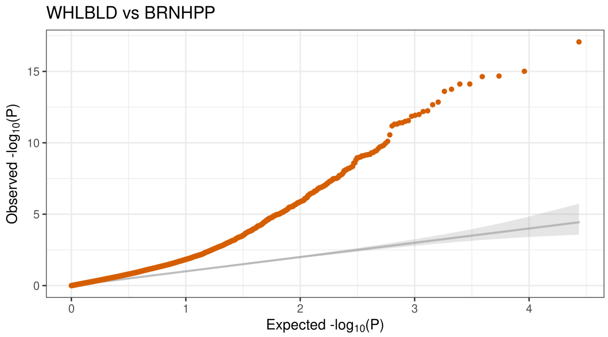
WHLBLD vs BRNHPP
Observed -log10(P)
Expected -log10(P)
0
5
10
15
0
1
2
3
4

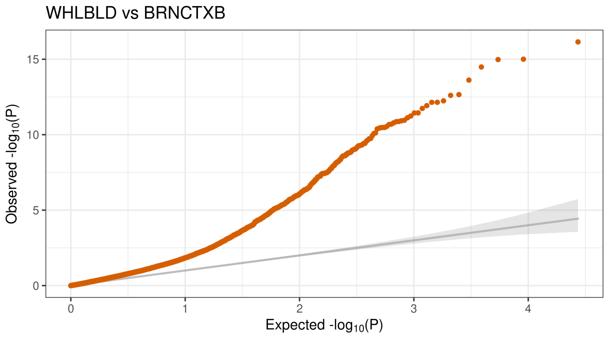
WHLBLD vs BRNCTXB
Observed -log10(P)
Expected -log10(P)
0
5
10
15
0
1
2
3
4

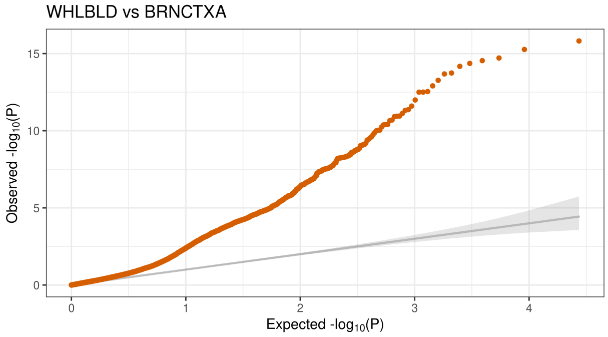
WHLBLD vs BRNCTXA
Observed -log10(P)
Expected -log10(P)
0
5
10
15
0
1
2
3
4

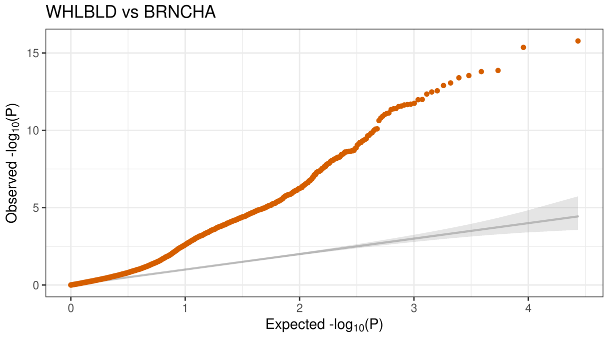
WHLBLD vs BRNCHA
Observed -log10(P)
Expected -log10(P)
0
5
10
15
0
1
2
3
4

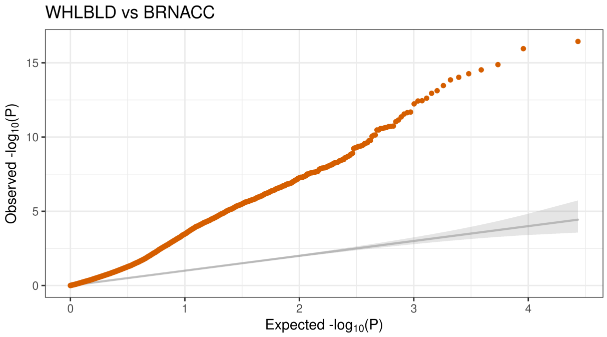
WHLBLD vs BRNACC
Observed -log10(P)
Expected -log10(P)
0
5
10
15
0
1
2
3
4
